# 873. Detection of Monkeypox Virus Using Microbial Cell-free DNA: Demonstrating the Potential of Agnostic Sequencing for Real Time Identification of an Emerging Pathogen

**DOI:** 10.1093/ofid/ofad500.918

**Published:** 2023-11-27

**Authors:** Martin S Lindner, Kevin Brick, Nicholas Noll, Sarah Y Park, Rachid Ounit, Asim Ahmed, Luis J Noa, Rabeeya Sabzwari, Ronald Trible, Jason Sniffen, Prerana J Roth, Amir Khan, Anamaria Rodriguez, Syeda Sahra, Michael J Davis, Inderjeet S Brar, Gayathri Balasundaram, Frederick S Nolte, Timothy A Blauwkamp, Sivan Bercovici

**Affiliations:** Karius, Inc., Mannheim, Baden-Wurttemberg, Germany; Karius, Redwood City, California; Karius, Redwood City, California; Karius, Redwood City, California; Karius, Redwood City, California; Karius, Redwood City, California; AdventHealth Orlando, Orlando, Florida; Edward Hines, Jr. VA Hospital, Hines, Illinois; Emory Saint Joseph's Hospital, Atlanta, Georgia; AdventHealth Orlando, Orlando, Florida; Prisma Health-Upstate, Greenville, South Carolina; Carle Foundation Hospital, Urbana, Illinois; Orlando Health, Orlando, Florida; Oklahoma University of Health Sciences Center, Oklahoma City, Oklahoma; University of Minnesota, DeWitt, Michigan; Baptist Memorial Hospital, Memphis, Tennessee; Karius, Redwood City, California; Karius, Redwood City, California; Karius Inc, Redwood City, California; Karius, Inc., Mannheim, Baden-Wurttemberg, Germany

## Abstract

**Background:**

The 2022 global monkeypox (mpox) outbreak has resulted in >86,000 cases worldwide. Mpox virus (mpoxv) PCR has failed for some divergent strains. Additionally, currently recommended testing requires a swab of lesions, which arise well after infection and may be unrecognized, especially in atypical disease. We present mpoxv detections using plasma microbial cell-free DNA (mcfDNA) sequencing.

**Methods:**

We characterized 13 case-patients in whom plasma mcfDNA sequencing, by the Karius CLIA certified/CAP accredited laboratory, detected and quantified the concentration of mpoxv in 16 samples. To confirm mpoxv detections, *in-silico* experiments were performed to test the inclusivity and exclusivity of the assay. Inclusivity was tested across the mpoxv genomes from the 2022 outbreak; exclusivity was tested to other *Poxviridae* viruses, some of which may be genetically similar to mpoxv, and other often co-occurring pathogens. In cases of sufficient sequencing coverage, mpoxv isolates were genotyped and phylodynamic information imputed using the nexstrain toolkit.

**Results:**

Case-patient characteristics at the time of plasma collection for mcfDNA sequencing are shown in Table 1. Mpox was not suspected in 6, with one having documented resolution of mpox >6 mos previously. For those with known mpox, mcfDNA sequencing was performed to determine other co-infections. Seven had moderate to severe mpox disease, supported by high mpoxv mcfDNA concentration; 4 died. Using mcfDNA coverage, 22 mutations were identified at 10 mpoxv genomic loci in 9 case-patients. 21 of the 22 variants were G >A or C >T, consistent with the predominant variation observed in the 2022 mpoxv outbreak. Phylogenetic comparison against public data facilitated mapping case-patient mpoxv strains to specific sublineages arising at different time points and associated with different geographic locations.

**Figure:**
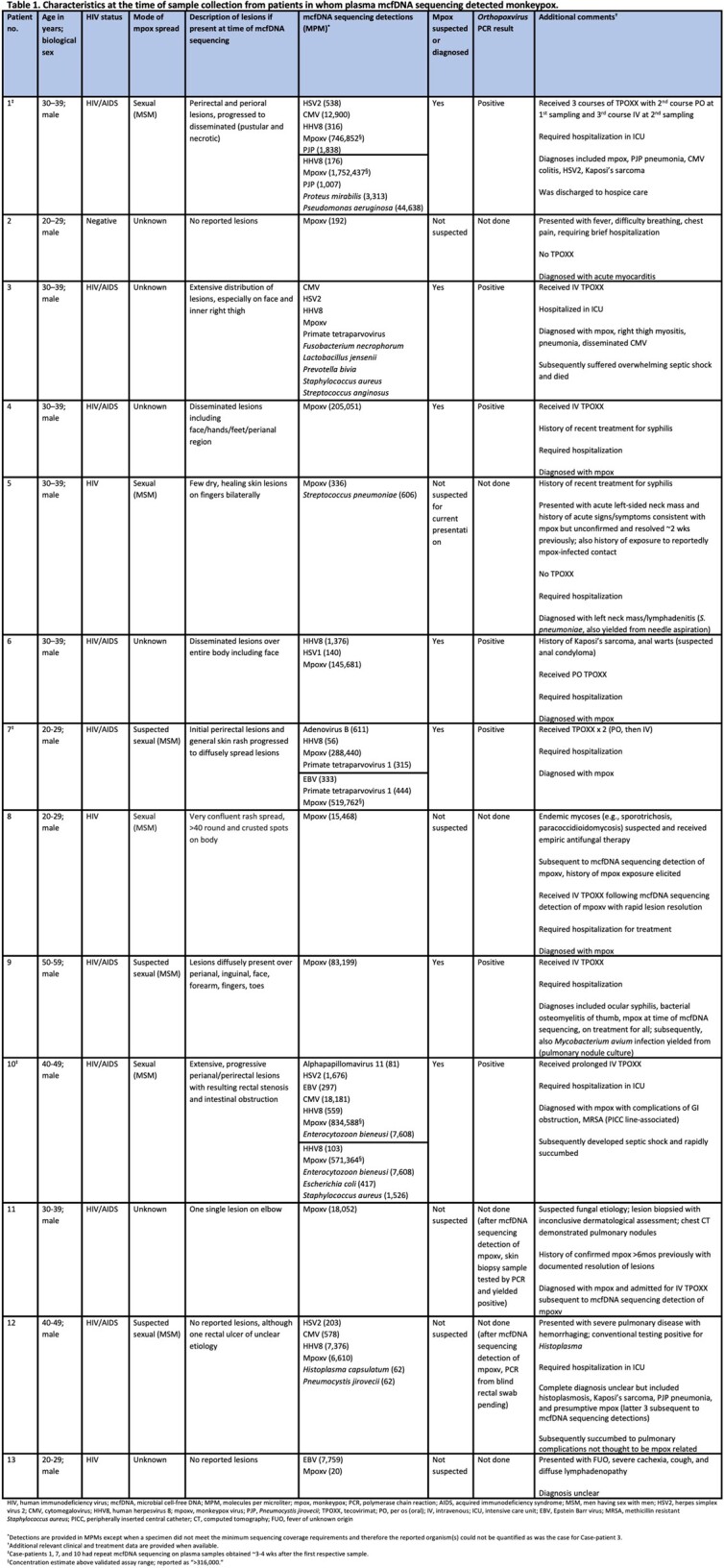

**Conclusion:**

We demonstrate the potential of plasma mcfDNA sequencing to detect, quantify, and subtype mpoxv using a single non-invasive test. This alternative to the current lesion-dependent PCR test may enhance disease surveillance and much needed situational awareness for public health agencies and scientists to track an emerging pathogen with potentially unexpected clinical presentations such as mpoxv.

**Disclosures:**

**Kevin Brick, PhD**, Karius: Employee|Karius: Stocks/Bonds **Nicholas Noll, Doctor of Philosophy**, Karius: Employee **Asim Ahmed, MD**, Karius: mcfDNA detection of infectious agents|Karius: Stocks/Bonds|Tarsus: Advisor/Consultant **Jason Sniffen, DO**, MicroGenDX: Advisor/Consultant **Gayathri Balasundaram, MSc**, Karius Inc: Employee|Karius Inc: Stocks/Bonds **Frederick S. Nolte, PhD**, Karius: Stocks/Bonds **Timothy A. Blauwkamp, PhD**, Karius: Board Member|Karius: Ownership Interest **Sivan Bercovici, PhD**, Karius: Stocks/Bonds

